# Life-long environmental enrichment counteracts spatial learning, reference and working memory deficits in middle-aged rats subjected to perinatal asphyxia

**DOI:** 10.3389/fnbeh.2014.00406

**Published:** 2015-01-05

**Authors:** Pablo Galeano, Eduardo Blanco, Tamara M. A. Logica Tornatore, Juan I. Romero, Mariana I. Holubiec, Fernando Rodríguez de Fonseca, Francisco Capani

**Affiliations:** ^1^Facultad de Medicina, Instituto de Investigaciones Cardiológicas “Prof. Dr. Alberto C. Taquini” (ININCA), Universidad de Buenos Aires (CONICET)Buenos Aires, Argentina; ^2^Instituto de Investigaciones Bioquímicas de Buenos Aires (CONICET), Fundación Instituto LeloirBuenos Aires, Argentina; ^3^Laboratorio de Investigación, Instituto de Investigación Biomédica (IBIMA), Universidad de Málaga - Hospital Regional Universitario de Málaga (UGC Salud Mental)Málaga, Spain; ^4^Departamento de Psicobiología y Metodología de las Ciencias del Comportamiento, Facultad de Psicología, Instituto de Investigación Biomédica (IBIMA), Universidad de MálagaMálaga, Spain

**Keywords:** perinatal asphyxia, environmental enrichment, aging, habituation, anxiety, recognition memory, spatial reference memory, spatial working memory

## Abstract

Continuous environmental stimulation induced by exposure to enriched environment (EE) has yielded cognitive benefits in different models of brain injury. Perinatal asphyxia results from a lack of oxygen supply to the fetus and is associated with long-lasting neurological deficits. However, the effects of EE in middle-aged rats suffering perinatal asphyxia are unknown. Therefore, the aim of the present study was to assess whether life-long exposure to EE could counteract the cognitive and behavioral alterations in middle-aged asphyctic rats. Experimental groups consisted of rats born vaginally (CTL), by cesarean section (C+), or by C+ following 19 min of asphyxia at birth (PA). At weaning, rats were assigned to standard (SE) or enriched environment (EE) for 18 months. During the last month of housing, animals were submitted to a behavioral test battery including Elevated Plus Maze, Open Field, Novel Object Recognition and Morris water maze (MWM). Results showed that middle-aged asphyctic rats, reared in SE, exhibited an impaired performance in the spatial reference and working memory versions of the MWM. EE was able to counteract these cognitive impairments. Moreover, EE improved the spatial learning performance of middle-aged CTL and C+ rats. On the other hand, all groups reared in SE did not differ in locomotor activity and anxiety levels, while EE reduced locomotion and anxiety, regardless of birth condition. Recognition memory was altered neither by birth condition nor by housing environment. These results support the importance of environmental stimulation across the lifespan to prevent cognitive deficits induced by perinatal asphyxia.

## Introduction

Perinatal asphyxia is an obstetric complication consisting in lack of oxygen supply to the fetus or newborn during a certain period of time (Adcock and Papile, 2008). Perinatal asphyxia has a worldwide incidence of about 1 per 1000 live births (McGuire, 2006) and is associated with a high mortality rate, neuropsychological impairments and increased risk to develop neurodevelopmental disorders, such as attention deficit hyperactivity disorder and schizophrenia (Lewis and Murray, 1987; Cannon et al., 2002; van Handel et al., 2007). Despite advances in medical technology, an effective treatment for perinatal asphyxia is lacking, nowadays.

To study perinatal asphyxia in experimental settings, two murine models have been developed: the so-called “Levine-Rice” model which is induced in 7-day-old rats by unilateral carotid ligation followed by oxygen deprivation (Rice et al., 1981) and the model developed by Bjelke et al. (1991) consisting in the immersion of the uterus horns still containing the fetuses, removed from ready-to-deliver rats, in a water bath at 37°C for 5–20 min (Capani et al., 2001). In the latter model, we have previously observed spatial reference and working memory impairments, increased astrogliosis and synaptic alterations in CA1 hippocampal area of adult rats (3–4 month-old) which had undergone 19 min of birth asphyxia (Saraceno et al., 2010, 2012; Galeano et al., 2011).

Although it has been suggested that brain alterations triggered by perinatal asphyxia could contribute to neurodegenerative disorders (Weitzdoerfer et al., 2004b), there is very scarce experimental data about the impact of perinatal asphyxia on cognitive domains in middle-aged and aged rats (Van de Berg et al., 2000; Weitzdoerfer et al., 2004a). Van de Berg et al. (2000) found an exaggerated age-related long-term memory impairment in 18-month-old perinatally asphyxiated rats, while Weitzdoerfer et al. (2002) reported that 24-month-old asphyctic rats showed a significantly longer escape latency during the re-learning trial in the Morris water maze compared with the matched age control group.

On the other hand, exposure of rodents to an enriched environment (EE) has consistently shown to improve memory and learning abilities in several behavioral tasks (Paylor et al., 1992; Rosenzweig and Bennett, 1996; van Praag et al., 2000; Duffy et al., 2001; Bruel-Jungerman et al., 2005; Leggio et al., 2005; Lores-Arnaiz et al., 2007), to prevent age-related memory impairments (Kempermann et al., 2002; Bennett et al., 2006; Lores-Arnaiz et al., 2006; Mora et al., 2007; Leal-Galicia et al., 2008; Obiang et al., 2011), to recover cognitive functions in murine models of hypoxia-isquemia (Dahlqvist et al., 2004; Komitova et al., 2006; Pereira et al., 2007, 2008, 2009; Sun et al., 2010), and to reduce spontaneous locomotion and anxiety-related behaviors (Falkenberg et al., 1992; Chapillon et al., 1999; Del Arco et al., 2007; Leal-Galicia et al., 2008; Segovia et al., 2008a,b; Hughes and Collins, 2010).

The cognitive improvement induced by EE was mainly associated with morphological and biochemical changes in the CA1 hippocampal area as well as an increase neurogenesis in the dentate gyrus (Iuvone et al., 1996; Ickes et al., 2000; Rampon et al., 2000; Nithianantharajah and Hannan, 2006). EE is a protocol in which groups of rats or mice are housed in bigger cages than those used as standard, with a more complex configuration, and where the animals are provided with different objects to explore that are changed frequently. In addition, enriched cages may have running wheels to allow animals to exercise. Thus, enriched cages provide the animals with physical, sensory, cognitive, and social stimulation that impact profoundly on brain structure and function (van Praag et al., 2000).

The main aim of the present study was to assess if life-long exposure to EE could counteract the cognitive alterations observed in middle-aged rats that had undergone 19 min of birth asphyxia. In addition, we assessed habituation and anxiety levels in middle-aged asphyctic rats and its modulation by EE. For this purpose, we chose classical tests that have traditionally been employed and widely validated in the literature to assess cognitive and emotional functions, such as the Elevated Plus Maze (EPM) test, the Open Field (OF) test, the Novel Object Recognition Task (NORT), and the spatial reference and working memory versions of the Morris water maze (MWM) test. Furthermore, we have previously employed these behavioral tasks in the global asphyxia model (with the exception of the NORT) in young animals (Galeano et al., 2011). Following this line of thought, we considered of value to investigate if cognitive and emotional deficits shown by young asphyctic rats worsen or remain the same through aging, and if these behavioral impairments could be counteracted by life-long exposure to environmental enrichment.

## Materials and methods

### Animals

Thirty-eight pregnant Sprague-Dawley rats were obtained from the animal care facilities at the School of Veterinary Sciences of the University of Buenos Aires and transferred to our local vivarium one week prior to delivery. Pregnant rats were housed individually in standard cages with *ad libitum* access to food and tap water. A total of 72 male offsprings of these pregnant rats were reared in standard (SE) or enriched (EE) environments from weaning to 18 months of age (see Section Housing Conditions). All animals were maintained in a temperature- (21 ± 2°C) and humidity- (50–60%) controlled environment on a 12 h light/dark cycle (lights on at 7:00 a.m.). All procedures were approved by the Institutional Animal Care and Use Committee of the School of Medicine at the University of Buenos Aires, in compliance with the Guide for the Care and Use of Laboratory Animals (NIH Publications No. 80-23, revised 1996). Every effort was made to minimize animal suffering and the number of animals used.

### Cesarean section and perinatal asphyxia procedures

We employed a murine model of perinatal asphyxia originally developed by Bjelke et al. (1991) and extensively described previously by our group (Capani et al., 1997, 2001, 2003, 2009; Saraceno et al., 2010, 2012; Galeano et al., 2011, 2013) with minor modifications. On gestational day 22, pregnant rats (*n* = 20) were left to deliver no more than two pups and were immediately euthanized by decapitation. Next, the uterus horns were rapidly isolated through an abdominal incision and one horn was opened, pups were removed, the amniotic fluid was cleaned, and the umbilical cord was ligated (cesarean section or C-section procedure). Concurrently, the remaining horn was placed in a water bath at 37°C for 19 min (moderate to severe perinatal asphyxia). Afterwards, the same procedures performed for the C-section were followed, but before ligation of the umbilical cord took place, pups were stimulated to breathe by performing tactile intermittent stimulation with pieces of medical wipes for a few minutes until regular breathing was established. This was unnecessary for pups born by C-section since they started breathing spontaneously. Male pups born by C-section (cesarean section group, C+; *n* = 24) or by C-section plus acute asphyxia (perinatal asphyxia group, PA; *n* = 24) were left to recover for approximately 1 h under a heating lamp. When the physiological conditions of the asphyxiated pups improved, C+ and PA pups were marked for identification and given to surrogate mothers who had delivered normally within the last 24 h. A ≈ 95% of survival rate was observed in those pups that were born by C-section. The survival rate dropped to ≈ 65% in those pups born by C-section plus acute asphyxia. Finally, another group of pregnant rats (*n* = 7) were left to deliver spontaneously and pups were cross-fostered among dams (vaginal delivery or control group, CTL; *n* = 24). In every case, we maintained litters of 10 pups with each mother. Only male pups were retained for all the subsequent procedures and studies.

### Housing conditions

**Figure 1:**
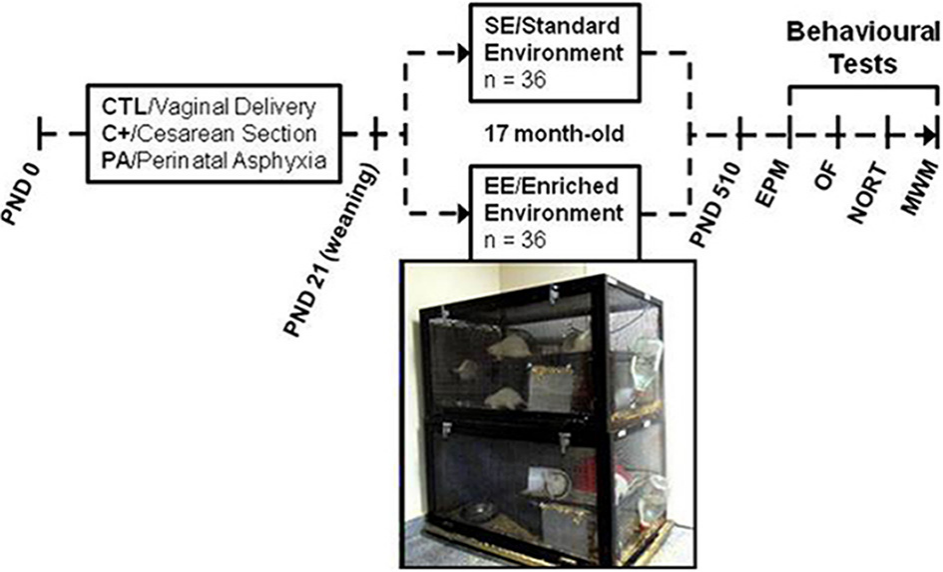
**Schematic diagram depicting study design and photograph of two of the enriched environment cages**. Cesarean section and perinatal asphyxia procedures were performed on gestational day 22 (PND day 0). At weaning (PND 21) rats from each birth condition (CTL, C+, and PA) were randomly assigned to standard (SE) or enriched environment (EE). After seventeen months, rats were assessed in a behavioral test battery including Elevated Plus Maze (EPM), Open Field (OF), Novel Object Recognition Test (NORT) and Morris water maze (MWM).

At weaning (PND 21) half of the rats of each group (12 CTL, 12 C+ and 12 PA) were housed in enriched environment (EE) cages (*n* = 6 rats per cage). The remaining rats in each group (12 CTL, 12 C+ and 12 PA) were housed in standard environment (SE) cages (*n* = 3 rats per cage). The EE consists of large cages (100 × 50 × 50 cm.) equipped with a floor platform (50 × 50 cm.), two inclined ramps, a chain hanging from the cage roof, two PVC tubes, two running wheels and five different safe toys of diverse materials (glass, metal, wood and plastic) that were changed every week (Figure 1). Cage walls were made of interwoven metal wires allowing rats to move in all directions. The SE consists of standard stainless steel cages of 29 × 21 × 34 cm. All animals were reared in SE or EE from weaning to 18 months of age.

### Behavioral studies

#### General procedures

At 17 months of age, all animals were submitted to behavioral assessment. Animals were naïve to all behavioral test procedures and were only manipulated during routine cage cleaning. Three days before Elevated Plus Maze test took place, all animals were handled once a day (5 min/day) and weighed. Behavioral procedures were carried out between 8:00 a.m. and 5:00 p.m. White noise was provided throughout testing. Testing order of the groups was counterbalanced to avoid the confounding effect of time of the day at which animals were tested. All training/testing sessions were recorded (JVC Everio GZ-HD620) and later analyzed using a computerized video-tracking system (Ethovision XT, version 9, Noldus Information Technology, Wageningen, The Netherlands) or the ethological observation software JWatcher V1.0.

#### Elevated Plus Maze test

The apparatus consisted of a black melamine central square platform (11 × 11 cm.) from which four black melamine arms radiated: two oppositely positioned open arms (50 × 11 × 0.25 cm.) and two oppositely positioned enclosed arms (50 × 11 × 40 cm.). The maze was elevated to a height of 100 cm. from the floor and indirectly illuminated (light intensity in open arms: 85–90 lux). Rats were placed individually into the central platform facing toward an open arm and allowed 5 min of free exploration of the apparatus. An arm entry was defined as the entry of all four paws into one arm. After each session the apparatus was cleaned with 70% ethanol. The following dependent variables were measured: total distance traveled, number of closed arm entries, percentage of open arm entries (open arm entries/total entries × 100) and percentage of time spent in open arms (time spent in open arms/300 × 100).

#### Open field (OF) test

The apparatus consisted in a square open-field arena made of black melamine (60 × 60 × 40 cm.). A central area of 30 × 30 cm. was arbitrarily defined and drawn over the image of the apparatus in the video-tracking system. A rat was considered to be into the central area when its four paws were on it. The arena was uniformly and indirectly illuminated (light intensity in the center of the OF: 70 lux). Rats were placed individually in the center of the arena and allowed 10 min of free exploration of the apparatus. Between sessions, the apparatus was cleaned with 70% ethanol. Dependent variables were: distance traveled, number of rears, time spent in the central area and the ratio central over total distance traveled (distance traveled in the center × 100/total distance traveled). Each variable was measured in two time bins of 5 min each.

#### Novel Object Recognition Task (NORT)

Although the Novel Object Recognition Task (NORT) is a behavioral test that was originally developed to assess non-spatial working memory (Ennaceur and Delacour, 1988), nowadays it is considered as a measure of the memory of an object (what), the location of an object (where) and the context in which the object was encountered (which) (Ennaceur, 2010). In the present study, this specific type of memory will be called “recognition memory.” Twenty-four hours after the OF test, the NORT took place in the same apparatus. Rats were presented with two identical objects and allowed to explore them for 5 min (sample trial). Animals were returned to their cages during the inter-trial interval. One hour later, one of the two familiar objects was replaced with a novel object and the rats were again allowed to explore them for 3 min (choice trial). Sets composed of three copies of the same object were used to prevent odor cues and all combinations and location of objects were used to prevent bias due to preference for a particular object or location. Exploration time was computed when the snout pointed to the object at a distance ≤ 2 cm. Discrimination index (d1) and discrimination ratio (d2) scores were calculated using the following formulas: d1 = tn – tf, and d2 = (tn – tf) / (tn + tf), where tn = the amount of time rats explored the novel object and tf = the amount of time rats explored the familiar object.

#### Morris water maze

***Apparatus.*** The water maze consisted of a circular black galvanized steel tank (180 cm. in diameter and 60 cm. deep). The tank was filled to a depth of 36–40 cm. with water at 22 ± 1°C. The maze was divided into four imaginary quadrants (A, B, C, and D) and a circular platform, made of transparent acrylic, was placed 2 cm above (visible escape platform) or beneath the water surface (hidden escape platform), in the center of one of the quadrants (35 cm. from the edge of the tank). To enhance the visibility of the platform during the cued learning training, a “flag” was attached to the platform. Four starting positions were stablished according to the four quadrants (a, b, c and d). To provide external reference points, multiple extra-maze visual cues of different shapes and sizes were hung on the wall of the experimental room. Indirect illumination was provided by four spiral compact fluorescent lamps in each corner facing the walls. Variables registered were: latency to find the escape platform, path length, and swimming speed.

***Cued learning.*** During cued learning the platform protruded 2 cm. above water surface and a “flag” was attached to it (visible escape platform). The maze was surrounded by black curtains to minimize the availability of extra-maze cues. For each of the four trials conducted on each day, the platform was moved to a different quadrant and a different start location was used. If a rat had not located the platform before 120 s elapsed, it was gently guided to the platform location and was allowed to remain there for 15 s. Inter-trial interval duration was approximately 30 s. Two days of cued training were conducted.

***Spatial learning and reference memory task.*** We used the same procedures described previously (Galeano et al., 2011, 2014) with some modifications. Briefly, the spatial learning task was conducted over five consecutive days with four trials per day. During each trial, a rat was gently released into the tank from one of the four starting positions and it was able to escape from the water using the hidden escape platform that was kept in the same location throughout the five sessions of the spatial learning task. A trial was finished when the rat found the escape platform or when 120 s had elapsed, whichever occurred first. If a rat failed to find the platform, the experimenter guided the animal to it. Rats remained on the platform for 15 s. Inter-trial interval duration was approximately 30 s. In each session, the four starting positions were used and the order of the sequence was changed pseudo-randomly between days. Twenty-four hours after the last trial of the spatial learning task, reference memory was assessed with a probe trial of 60 s in which the escape platform was removed from the tank and each rat was released from a new starting position not used during the spatial learning task. Time spent in each quadrant was recorded. When sessions finished rats were dried and returned to their home cage.

***Spatial working memory task.*** Forty-eight hours after the probe trial, rats were submitted to a spatial working memory task over five consecutive days. Each rat was given two trials per day: a sample and a retention trial with a 30 s inter-trial interval during which the rat remained in its transport cage. In both trials, each rat was gently released into the tank from one of the four starting positions and allowed to locate the hidden escape platform up to a maximum of 90 s. When rats failed to find the platform, the experimenter guided the animal to it. Rats remained on the platform for 15 s. Starting points and location of the platform were pseudo-randomly varied for each rat throughout the 5 days but fixed within a single session. In this manner, rats have to hold the information about the location of the platform during the sample trial easily available to find again the platform in the retention trial as efficiently as possible, being useless the information from previous days. Starting points and platform were never at the same quadrant and neither the platform location nor the starting point was the same as from the previous day. For more details about the procedure see Galeano et al. (2011).

### Statistical analysis

The results were expressed as the mean ± s.e.m. Data were analyzed by ANOVA tests followed by Tukey's *post hoc* tests for multiple comparisons, unless noted otherwise. The null hypothesis was rejected when the two tailed probability value was 5% or less (*p* ≤ 0.05). All statistical analyses were performed using the SPSS 15.0 for windows (SPSS Inc., Chicago, IL, USA).

## Results

Since interactions between birth condition (CTL, C+, and PA) and housing environment (SE and EE) were found in the spatial reference and working memory versions of the MWM, but in none of the other tests, results are described and discussed in the following order: Morris water maze, Elevated Plus Maze test, Open Field test and Novel Object Recognition Task.

### Cue learning in the morris water maze

Three-Way mixed ANOVA test carried out on the latency to the visible escape platform showed that the main effect of day was significant [*F*_(1, 66)_ = 58.34, *p* < 0.001]. Neither the remaining main effects nor any of the interactions resulted to be significant (*F* ≤ 1 for all cases). These results suggest that neither of the groups were visually or motivationally impaired.

### Spatial learning in the morris water maze

The results of the Three-Way mixed ANOVA tests (see Table 1) indicated that rats displayed a progressive reduction in escape latencies and path lengths across the five days of learning, but the rates of reduction were not similar between groups, since most of the interactions were significant (see Table 1). To determine if groups differ in their escape latencies and path lengths in specific days of the spatial learning task, One-Way ANOVA tests were conducted. On days 2, 3, 4, and 5 One-Way ANOVA tests were significant for both variables (see Table 2).

**Table 1:** **Statistical results of the Three-Way mixed ANOVA tests performed on data from the spatial learning phase of the reference memory version of the Morris water maze**.

**Main effects and interactions**	**Escape latency**	**Path length**
Environment	*F*_(1, 66)_ = 65.35, *p* < 0.001	*F*_(1, 66)_ = 93.19, *p* < 0.001
Birth condition	*F*_(2, 66)_ = 37.69, *p* < 0.001	*F*_(2, 66)_ = 43.88, *p* < 0.001
Day	*F*_(2.49, 164.59)_ = 227.8, *p* < 0.001	*F*_(2.84, 187.77)_ = 299.03, *p* < 0.001
Environment × birth condition	*F*_(2, 66)_ = 2.56, *p* = 0.085	*F*_(2, 66)_ = 3.40, *p* = 0.039
Environment × day	*F*_(2.49, 164.59)_ = 8.28, *p* < 0.001	*F*_(4.98, 164.59)_ = 3.03, *p* = 0.03
Birth condition × day	*F*_(2.84, 187.77)_ = 11.02, *p* < 0.001	*F*_(5.69, 187.77)_ = 3.80, *p* < 0.01
Environment × birth condition × day	*F* < 1, *p* = n.s.	*F* < 1, *p* = n.s.

**Table 2:** **Statistical results of One-Way ANOVA tests performed on data from each day of the spatial learning phase of the reference memory version of the Morris water maze**.

**Main effect**	**Escape latency**	**Path length**
Day 1	*F* < 1, *p* = n.s.	*F* < 1, *p* = n.s.
Day 2	*F*_(5, 66)_ = 26.29, *p* < 0.001	*F*_(5, 66)_ = 33.94, *p* < 0.001
Day 3	*F*_(5, 66)_ = 27.73, *p* < 0.001	*F*_(5, 66)_ = 29.83, *p* < 0.001
Day 4	*F*_(5, 66)_ = 29.86, *p* < 0.001	*F*_(5, 66)_ = 35.68, *p* < 0.001
Day 5	*F*_(5, 66)_ = 3.78, *p* = 0.005	*F*_(5, 66)_ = 3.73, *p* = 0.005

**Figure 2:**
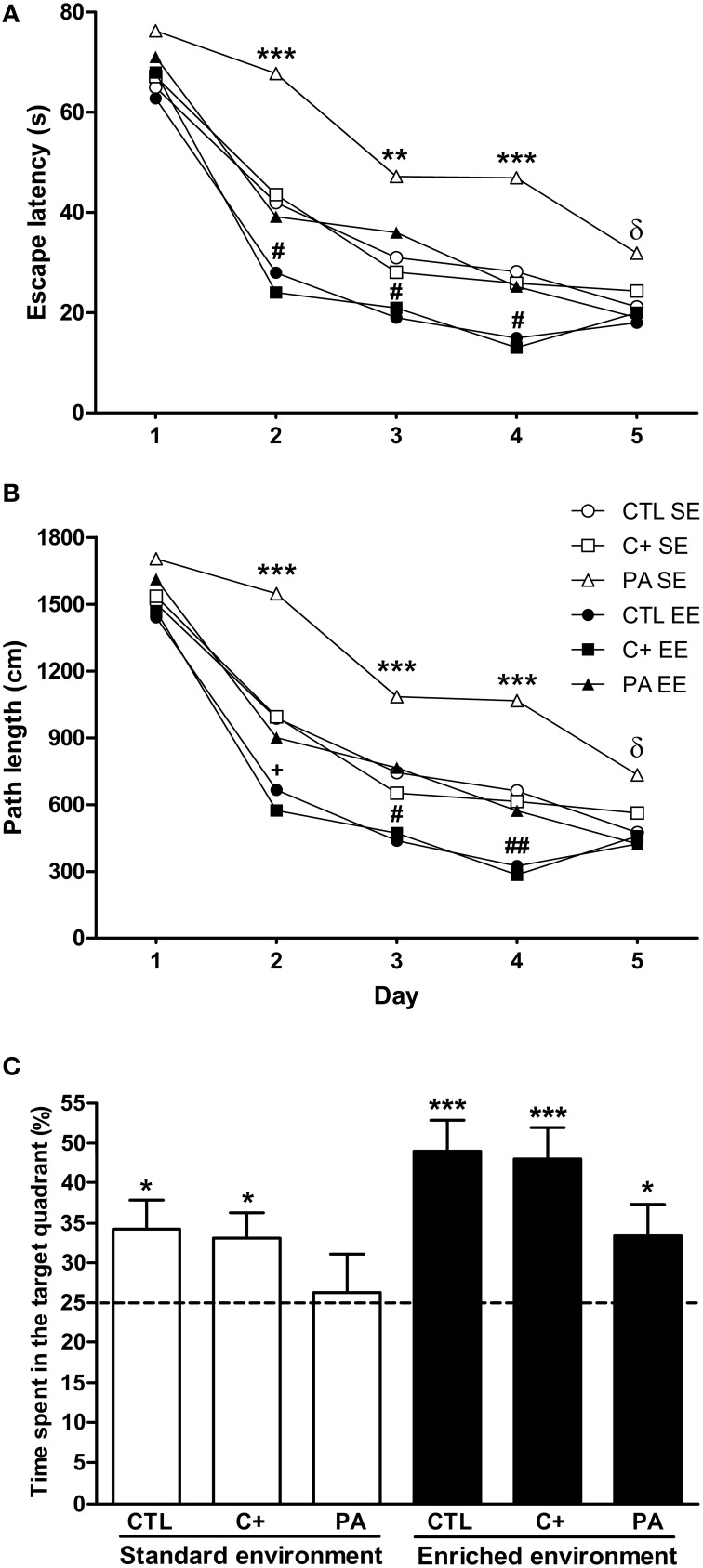
**Enriched environment was able to counteract the spatial learning and reference memory impairment displayed by middle-aged asphyctic rats**. **(A)** Escape latency; **(B)** Path length; **(C)** Time spent in the target quadrant. CTL SE, vaginal delivery rats reared in standard environment; C+ SE, rats born by cesarean section reared in standard environment; PA SE, rats born by cesarean section + asphyxia reared in standard environment; CTL EE, vaginal delivery rats reared in enriched environment; C+ EE, rats born by cesarean section reared in enriched environment; PA EE, rats born by cesarean section + asphyxia reared in enriched environment; CTL, vaginal delivery rats; C+, rats born by cesarean section; PA, rats born by cesarean section + asphyxia. In **(C)** data are expressed as the mean + s.e.m. of *n* = 12. In **(A,B)** error bars are omitted for clarity. ^*^p < 0.05 vs. time expected by chance **(C)**; ^**^p < 0.01 PA SE vs. all other groups **(A,B)**; ^***^p ≤ 0.001 PA SE vs. all other groups **(A,B)** or vs. time expected by chance **(C)**; ^δ^*p* < 0.05 PA SE vs. CTL, C+ and PA reared in EE **(A,B)**; ^+^*p* < 0.05 CTL EE or C+ EE vs. all other groups except PA reared in EE (*p* = 0.065) **(A,B)**; ^#^*p* < 0.05 CTL EE or C+ EE vs. all other groups (a and b); ^##^*p* < 0.01 CTL EE or C+ EE vs. all other groups **(A,B)**.

*Post-hoc* multiple comparisons revealed that PA rats reared in SE showed the worst performance with the highest escape latencies and the longest path lengths from day 2 to 4 (Figures 2A,B). In addition, on day 5 PA rats reared in SE showed higher escape latencies and longer path lengths in comparison to rats reared in EE (Figures 2A,B). CTL and C+ rats reared in EE displayed the best performance in the spatial learning task with the lowest escape latencies and the shortest path lengths from day 2 to 4 (Figures 2A,B). PA rats reared in EE showed similar escape latencies and path lengths to those showed by CTL and C+ rats reared in SE, displaying the three groups an intermediate performance (Figures 2A,B). These differences in spatial learning abilities could not be ascribed to sensorimotor or motivational differences associated with birth condition or housing environment since swimming speed was similar among the groups (see Supplementary Material 1). These results indicate that the life-long exposure to an EE improved spatial learning abilities in middle-aged rats. Furthermore, EE was able to counteract the impairment in spatial learning abilities showed by PA rats reared in SE.

### Spatial reference memory in the morris water maze

To analyze the performance of animals in the probe trial, Two-Way ANOVA test was carried out. Results showed that the main effects of environment and birth condition were significant [*F*_(1, 66)_ = 7.76, *p* = 0.007; *F*_(2, 66)_ = 3.35, *p* = 0.041, respectively], but the interaction environment × birth condition was not (*F* < 1). *Post-hoc* pairwise comparisons indicated that groups did not differ from each other in the time spent in the target quadrant during the probe trial, although a strong tendency was detected for CTL and C+ rats reared in EE in comparison with PA rats reared in SE (*p* = 0.067 and *p* = 0.085, respectively). To further analyze the performance of groups during the probe trial, one-sample *t*-tests were carried out. Results indicated that all groups, but PA reared in SE, spent a significantly longer time than expected by chance (15 s) in the quadrant where the escape platform was located during the spatial learning training (CTL SE: *t* = 2.58, *p* = 0.026; C+ SE: *t* = 2.59, *p* = 0.025; PA SE: *t* = 0.029, *p* = n.s.; CTL EE: *t* = 4.94, *p* < 0.001; C+ EE: *t* = 4.60, *p* = 0.001; PA EE: *t* = 2.32, *p* = 0.037) (Figure 2C). These results indicated that life-long exposure to EE was able to counteract the reference memory deficit shown by middle-aged asphyctic rats reared in SE.

### Spatial working memory in the morris water maze

**Figure 3:**
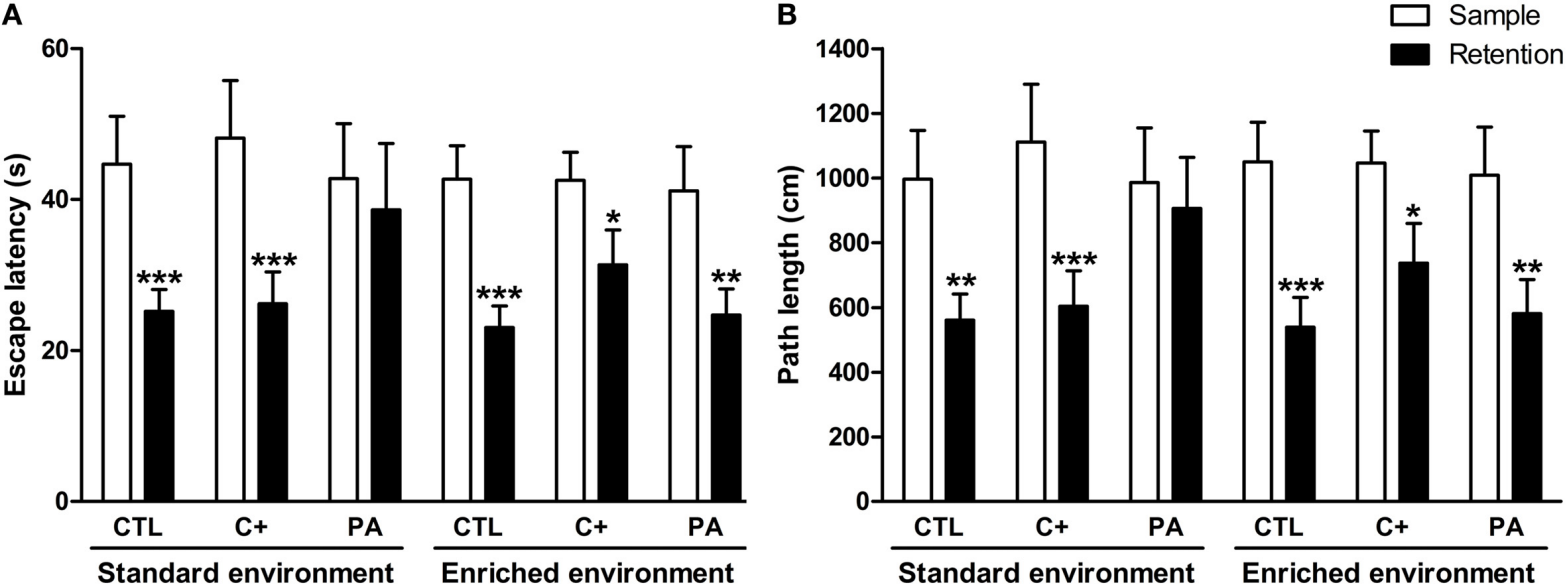
**Spatial working memory impairment displayed by middle-aged asphyctic rats was counteracted by life-long exposure to environmental enrichment**. **(A)** Escape latency; **(B)** Path length. CTL, vaginal delivery rats; C+, rats born by cesarean section; PA, rats born by cesarean section + asphyxia. Each bar represents the mean + s.e.m. of *n* = 12. ^*^p < 0.05; ^**^p ≤ 0.01; ^***^p ≤ 0.001 compared to sample trials.

Three-Way mixed ANOVA tests carried out on escape latencies and path lengths showed that the main effects of type of trial (sample and retention) were significant for both variables [*F*_(1, 66)_ = 45.29, *p* < 0.001; *F*_(1,66)_ = 42.77, *p* < 0001, respectively]. Neither of the remaining main effects nor any of the interactions were significant for either variable (*p* = n.s. for all cases). When escape latencies and path lengths were further analyzed by *post-hoc* pairwise comparisons, it was revealed that PA rats reared in SE did not show a significant reduction in escape latencies or in path lengths during the retention trials in comparison with those displayed during the sample trials (*p* = n.s. for both variables), while all the remaining groups did (*p* < 0.05 in all cases) (Figures 3A,B). No differences in swimming speed were found (see Supplementary Material 2). These results mean that middle-aged asphyctic rats reared in SE displayed a spatial working memory impairment that was counteracted by EE.

### Locomotor activity and anxiety-related behaviors in the EPM test

**Figure 4:**
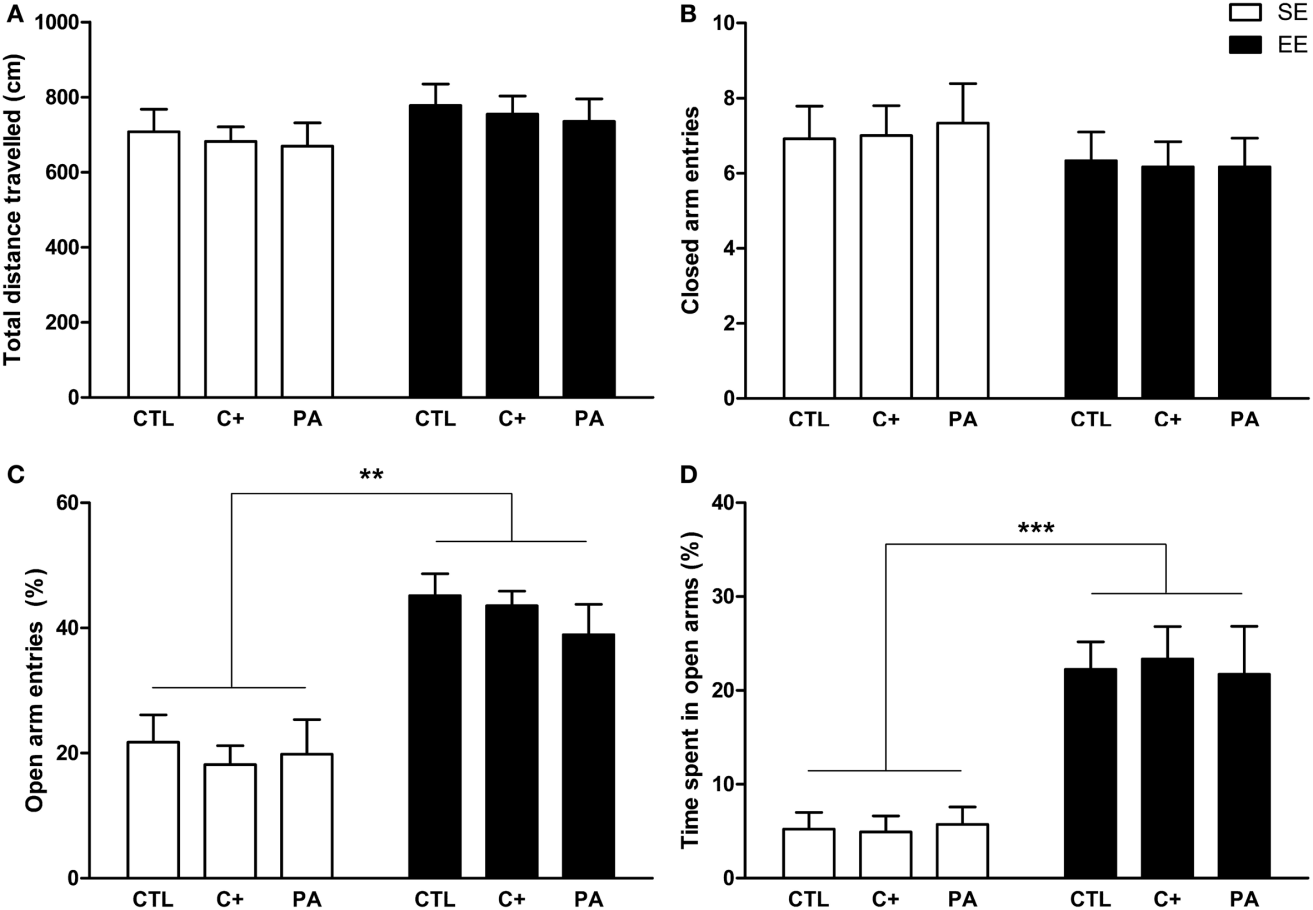
**Middle-aged rats reared in an enriched environment showed reduced anxiety-related behaviors in the Elevated Plus Maze test**. **(A)** Total distance traveled; **(B)** number of closed arm entries; **(C)** percentage of open arms entries; **(D)** percentage of time spent in open arms. CTL, vaginal delivery rats; C+, rats born by cesarean section; PA, rats born by cesarean section + asphyxia; SE, standard environment; EE, enriched environment. Each bar represents the mean + s.e.m. of *n* = 12. ^**^p < 0.01; ^***^p < 0.001.

The Two-Way ANOVA tests performed on total distance traveled and closed arm entries indicated that neither the main effects of environment [*F*_(1, 66)_ = 2.40, *p* = n.s.; *F*_(1, 66)_ = 1.62, *p* = n.s., respectively] nor the main effects of birth condition (*F* < 1 for both variables) nor the interactions environment × birth condition (*F* < 1 for both variables) were significant (Figures 4A,B). Regarding the percentage of open arm entries and the percentage of time spent in open arms, the main effects of environment were significant for both variables [*F*_(1, 66)_ = 46.15, *p* < 0.001; *F*_(1, 66)_ = 47.07, *p* < 0.001, respectively], but the main effects of birth condition and the interactions environment x birth condition were not significant for either variable (*F* < 1 in every case) (Figures 4C,D). *Post-hoc* pairwise comparisons confirmed that the CTL, C+, and PA rats reared in EE showed a significantly higher percentage of open arm entries (*p* < 0.01 for all the comparisons) and a significantly higher percentage of time spent in open arms (*p* < 0.001 for all the comparisons) compared to the CTL, C+, and PA rats reared in SE (Figures 4C,D). These results indicate that middle-aged rats reared in EE displayed reduced anxiety-related behaviors regardless of birth condition (Figures 4C,D), while no differences were observed in locomotor activity (Figures 4A,B).

### Locomotion, exploratory activity and anxiety-related behaviors in the of test

**Figure 5:**
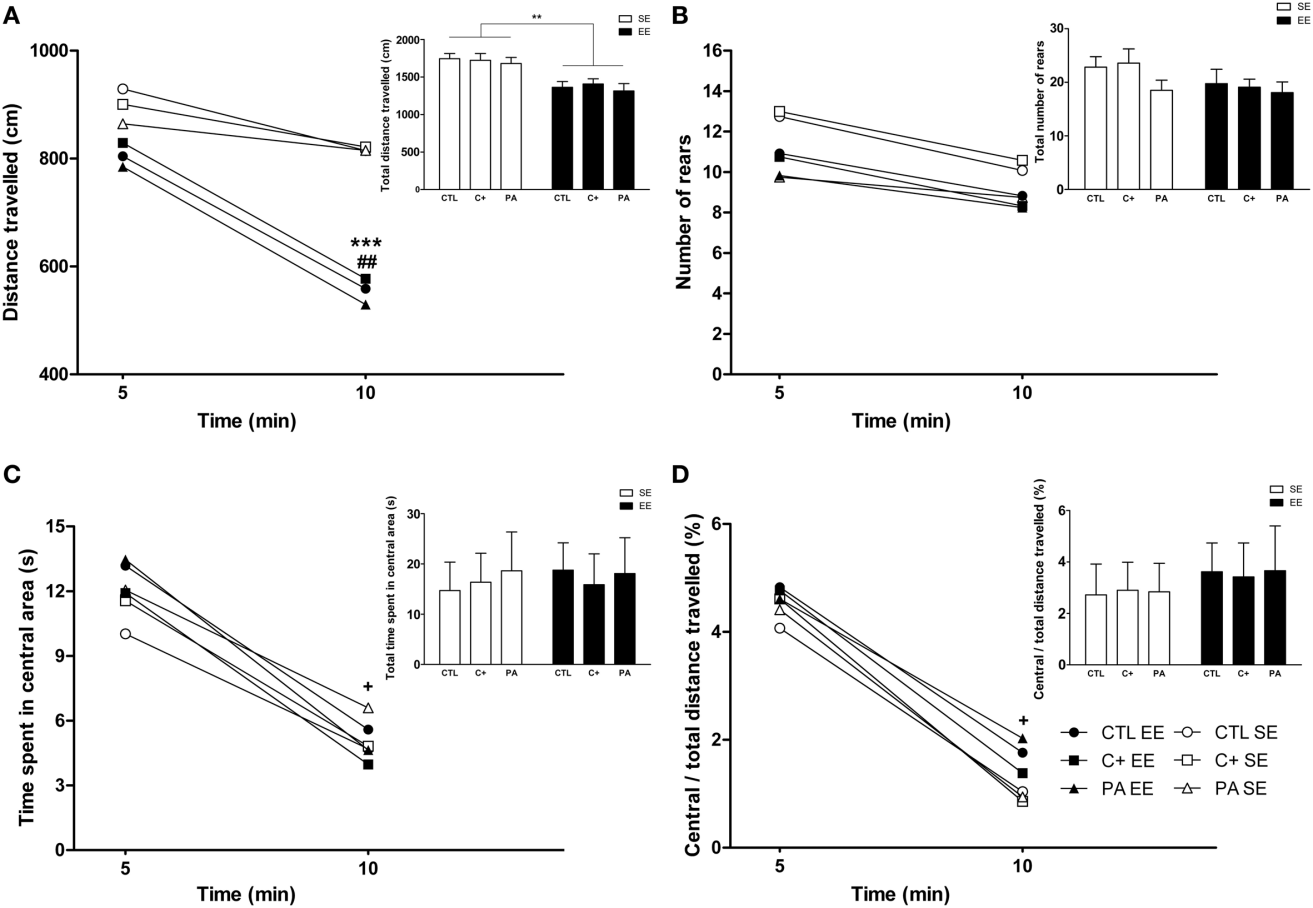
**Middle-aged rats reared in an enriched environment displayed rapid habituation to the Open Field test**. **(A)** Distance traveled over time, insert: total distance traveled; **(B)** number of rears over time, insert: total number of rears; **(C)** time spent in the central area over time, insert: total time spent in the central area; **(D)** ratio central / total distance traveled over time, insert: Ratio central / total distance traveled during the entire OF session. CTL SE, vaginal delivery rats reared in standard environment; C+ SE, rats born by cesarean section reared in standard environment; PA SE, rats born by cesarean section + asphyxia reared in standard environment; CTL EE, vaginal delivery rats reared in enriched environment; C+ EE, rats born by cesarean section reared in enriched environment; PA EE, rats born by cesarean section + asphyxia reared in enriched environment; CTL, vaginal delivery rats; C+, rats born by cesarean section; PA, rats born by cesarean section + asphyxia; SE, standard environment; EE, enriched environment. Data are expressed as the mean **(A–D)** or the mean + s.e.m. [inserts] of *n* = 12. In **(A–D)** error bars are omitted for clarity. ^***^p < 0.001 and ^**^p < 0.01 compared to groups reared in SE, ^##^*p* < 0.01 and ^+^*p* < 0.05 compared to the first 5 min time bin.

The Three-Way mixed ANOVA test carried out on the distance traveled showed that the main effect of environment, time bin and the interaction time bin × environment were significant [*F*_(1, 66)_ = 28.76, *p* < 0.001; *F*_(1, 66)_ = 47.29, *p* < 0.001; *F*_(1, 66)_ = 12.39, *p* = 0.001, respectively]. The remaining main effects and interactions did not reach statistical significance (*F* < 1 in every case). *Post-hoc* pairwise comparisons revealed that during the first 5 min of exploration the distance traveled was similar among the groups (*p* = n.s. for all the comparisons) (Figure 5A). On the contrary, during the second 5 min of exploration the distance traveled by groups reared in EE was significantly shorter than for groups reared in SE (*p* < 0.001 for all the comparisons) (Figure 5A). Paired *t*-tests confirmed that CTL, C+ and PA rats reared in EE significantly reduced their locomotor activity between the first and the second 5 min time bin (*t* = 4.08, *df* = 11, *p* = 0.002; *t* = 4.30, *df* = 11, *p* = 0.001; *t* = 5.14, *df* = 11, *p* < 0.001, respectively), while CTL, C+ and PA rats reared in SE did not (*p* = n.s. for each paired *t*-test) (Figure 5A). When distance traveled was collapsed across time bins, the Two-Way ANOVA test showed that the main effect of environment was significant [*F*_(1, 66)_ = 28.76, *p* < 0.001], but the main effect of birth condition and the interaction environment x birth condition were not (*F* < 1 for both cases). *Post-hoc* pairwise comparisons confirmed that groups reared in EE displayed a significantly shorter total distance traveled than groups reared in SE (*p* < 0.01 for all the comparisons) (see insert in Figure 5A). This reduction in the total distance traveled is attributable to the reduction in the distance traveled displayed during the second 5 min time bin. When the Three-Way mixed ANOVA test was performed on the number of rears, the time bin was the only effect that resulted to be significant [*F*_(1, 66)_ = 17.02, *p* < 0.001]. This means that when data from all groups were pooled, rats displayed, during the second 5 min time bin, a significantly lower number of rears than during the first 5 min time bin. However, paired *t*-tests revealed that none of the groups individually displayed significant changes in the number of rears between the first and the second 5 min time bin (*p* = n.s. for each paired *t*-test) (Figure 5B). When the number of rears was collapsed across time bins, the Two-Way ANOVA test showed that neither the main effect of environment nor the main effect of birth condition nor the interaction environment x birth condition were significant [*F*_(1, 66)_ = 2.29, *p* = n.s.; *F*_(2, 66)_ = 1.30, *p* = n.s.; *F* < 1, respectively] (see insert in Figure 5B). Regarding the time spent in the central area and the ratio central over total distance traveled, the Three-Way mixed ANOVA tests indicated that the main effects of time bin were significant for both variables [*F*_(1, 66)_ = 46.17, *p* < 0.001; *F*_(1, 66)_ = 36.92, *p* < 0.001, respectively], while no other main effects nor any of the interactions reached statistical significance (*p* = n.s. in every case). Paired *t*-tests confirmed that all groups, regardless of the environment or the birth condition, spent significantly less time in the central area and showed a significantly lower ratio central over total distance traveled during the second 5 min time bin than during the first 5 min time bin (*p* < 0.05 for all paired *t*-tests) (Figures 5C,D). Finally, when data were collapsed, the Two-Way ANOVA tests performed on both variables indicated that neither the main effects of environment nor the main effects of birth condition nor the interactions environment x birth condition were significant (*F* < 1 for every case) (see inserts in Figures 5C,D).

Overall, these results indicate that middle-aged rats reared in EE, regardless of birth condition, habituated their horizontal locomotor activity but not their vertical locomotor activity (rearing) when exposed to the OF (Figures 5A,B). Neither the environment nor the birth condition seemed to affect anxiety-related behaviors in this test (see Figures 5C,D and inserts in both figures).

### Recognition memory in the NORT test

**Figure 6:**
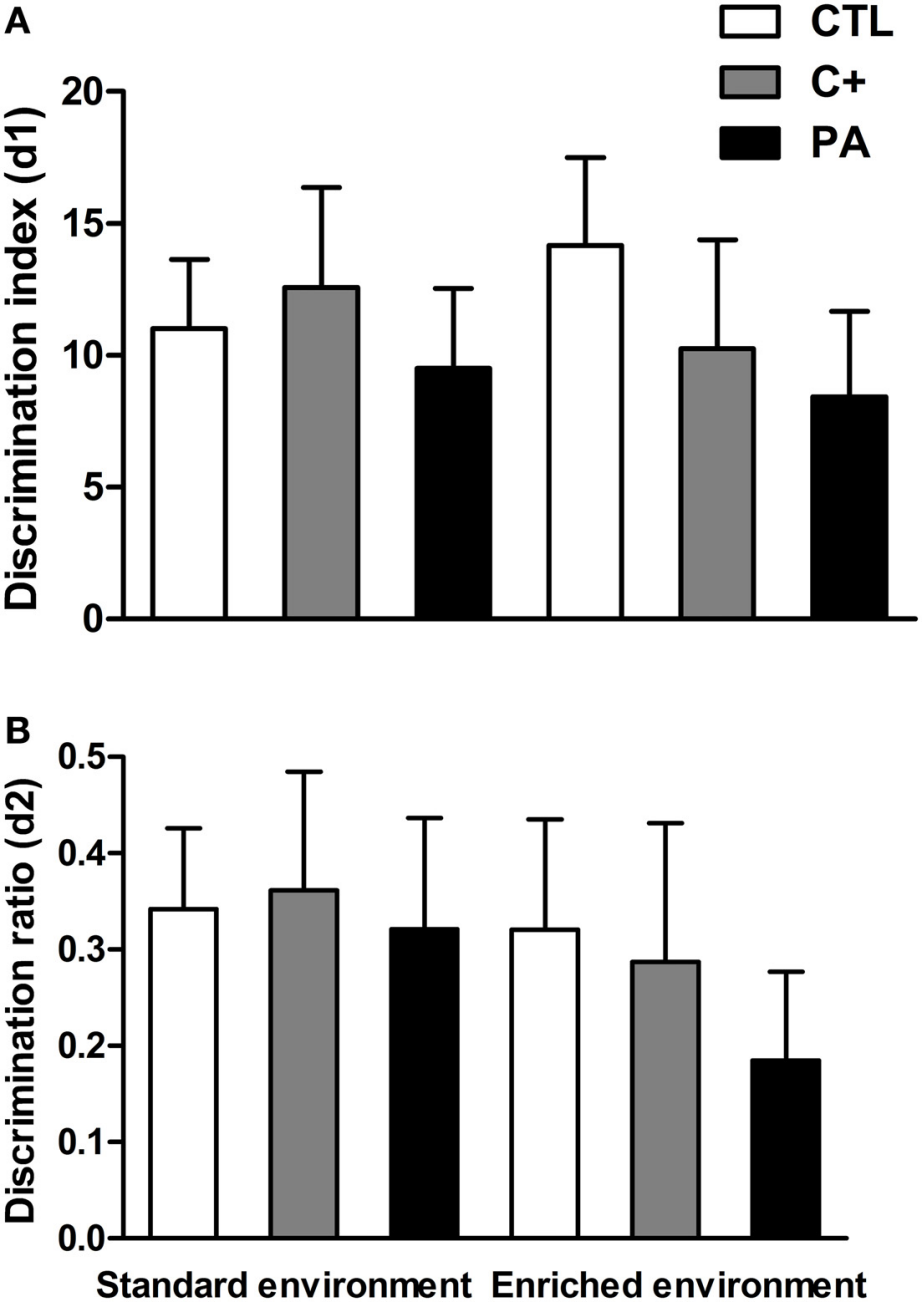
**Neither environment nor birth condition did affect the performance of rats in the Novel Object Recognition test**. **(A)** discrimination index (d1); **(B)** discrimination ratio (d2). All groups showed preference for the novel object, spending more time exploring it. CTL: vaginal delivery rats; C+: rats born by cesarean section; PA, rats born by cesarean section + asphyxia. Each bar represents the mean + s.e.m. of *n* = 12.

Two-Way ANOVA tests performed on discrimination index (d1) and discrimination ratio (d2) scores indicated that neither the main effects of environment nor the main effects of birth condition nor the interactions environment × birth condition were significant for either of the two variables (*F* < 1 for all cases) (Figures 6A,B). Thus, recognition memory was not deteriorated by aging or by the synergy between aging and asphyxia. Besides, EE was not able to improve recognition memory in either of the groups.

## Discussion

The present study investigated the effects of life-long exposure to EE in middle-aged rats that were subjected to 19 min of asphyxia at birth. Our aim was to determine whether perinatal asphyxia worsens age-induced cognitive and behavioral impairments. Moreover, we studied whether continuous exposure to EE from weaning (21 post-natal day) to 18 months of age improves cognitive and emotional performance. To address these issues, rats born vaginally, by cesarean section or by cesarean section with acute anoxia, were reared until the age of 18 months in SE or EE and evaluated, during the last month of the housing protocol, in a behavioral test battery that included the following tests: Elevated Plus Maze (EPM) test, Open Field (OF) test, Novel Object Recognition Test (NORT) and the spatial reference and working memory versions of the Morris water maze (MWM). Since interactions between birth condition and housing environment were only found in the MWM test, the results from this task are discussed firstly.

It is well known that birth injuries, such as perinatal asphyxia, may cause long-lasting cognitive dysfunctions in adulthood (Galeano et al., 2011). Our data showed that middle-aged asphyctic rats displayed impaired spatial learning, reference and working memory in the MWM (see Figures 2, 3). It is important to note that during the first day of spatial learning no differences between groups were observed. This suggests a specific retention impairment in asphyctic animals, while the codification process remains unaltered. To the contrary, Van de Berg et al. (2000) and Weitzdoerfer et al. (2002) did not find differences between middle-aged and aged control and asphyctic rats during the learning phase and the retention probe trial in the MWM. Several methodological differences could account for these discrepancies, although the main one could be the smaller tanks used in both studies in comparison with the one employed in the present work (122 and 140 cm. in diameter in the studies by Van de Berg et al. (2000) and Weitzdoerfer et al. (2002), respectively, compared with 180 cm. in diameter in this study). Performance in the MWM showed to be highly sensitive to variations in the diameter of the apparatus (Vorhees and Williams, 2006). Smaller tanks could make the task more easily solved and thus mask differences between groups. In addition, both studies used different protocols from the one employed in this study. Van de Berg et al. (2000) submitted middle-aged rats to a two-day protocol (two trials per day), while Weitzdoerfer et al. (2002) employed a four-day protocol with the last day of spatial learning conducted 10–14 days later from the first three ones. Furthermore, in the latter study, only female rats were used as experimental subjects. Nevertheless, in this same study when the platform was relocated to the opposite quadrant, 10–14 days after the last learning trial, aged asphyctic rats showed an impaired ability to re-learn the new platform location. This could be interpreted as a deficit of cognitive flexibility in aged asphyctic rats. In summary, from the results obtained by Weitzdoerfer et al. (2002) and by us, it could be concluded that the maturation process in rats which suffered from perinatal asphyxia at birth could induce cognitive alterations consisting mainly of spatial learning deficits, spatial long-term memory alterations, impaired cognitive flexibility and spatial working memory disturbances. In this sense, neurological deficits induced by perinatal asphyxia may aggravate the cognitive decline induced by the aging process.

Environmental enrichment could alleviate the cognitive alterations and lead to an improvement of spatial memory performance (Kempermann et al., 2002; Sampedro-Piquero et al., 2013, 2014a,b). In this sense, in the present study we showed that a prolonged exposure to EE had a beneficial effect *per se*, since control groups reared in EE (CTL EE and C+ EE) exhibited a better performance in the spatial learning phase compared to control animals reared in SE (CTL SE and C+ SE) (see Figures 2, 3). This beneficial effect of extensive EE on learning process is consistent with previous results obtained by other groups (Leggio et al., 2005; Lores-Arnaiz et al., 2006). Thus, life-long exposure to EE may prevent or delay the cognitive impairments associated with normal aging. In addition, life-long exposure to EE was able to recover the spatial learning, reference and working memory deficits displayed by middle-aged asphyctic rats reared in SE. To our knowledge, this is the first study that demonstrates a beneficial effect of life-long exposure to EE on cognitive impairments shown by middle-aged asphyctic rats. There are some other studies that have evaluated the effects of EE on cognitive deficits induced by neonatal anoxia (Iuvone et al., 1996) or neonatal hypoxia-ischemia (Pereira et al., 2007, 2008). For example, Pereira et al. (2007, 2008) reported that daily exposure (1 h/day for 9 weeks) or continuous housing in an EE (from postnatal days 8–30) recovered spatial memory deficits in the model of brain damage induced by neonatal hypoxia-ischemia. Although these studies were conducted using a different model of perinatal asphyxia in young animals, it suggests that in both models of perinatal asphyxia (the one developed by Bjelke et al., 1991 and that developed by Rice et al., 1981) exposure to EE prevents the spatial reference and working memory deficits induced by the birth insult.

Regarding anxiety and locomotion, our results showed that middle-aged rats subjected to perinatal asphyxia at birth, and reared in SE, did not differ in anxiety-related behaviors (open arm entries and time spent in open arms in the EPM; time spent in central area and central/total distance traveled in the OF) and locomotor activity (distance traveled and closed arm entries in the EPM; distance traveled in the OF) compared to CTL and C+ rats also reared in SE (see Figures 4, 5). We have previously reported that perinatal asphyxia is not associated with increased anxiety-related behaviors during adulthood (3-month-old asphyctic rats) (Galeano et al., 2011). Therefore, our data seem to indicate that perinatal asphyxia is not linked to increased anxiety across the lifespan. In contrast to the results obtained for anxiety-related behaviors, we have also reported that perinatal asphyxia led to a significant reduction in locomotion and in novelty exploration during young adulthood, whereas young adult rats born by cesarean section only displayed a mild deficit in novelty exploration (Galeano et al., 2011). In the present study, middle-aged asphyctic rats, reared in SE, showed similar levels of locomotor activity to middle-aged CTL and C+ rats reared in the same environment (see Figures 4A,B, 5A,B). This lack of differences could be explained by an age-induced reduction in motivation to explore new environments in control rats and thus, to a possible floor effect. Moreover, horizontal locomotor activity is lower in middle-aged rats than in young adult rats, regardless of the birth condition, since the distance covered in the OF in the present study was much lower than in the previous cited report (Galeano et al., 2011) (taking into account only the first 10 min of the OF test duration of the previous report). These results are in agreement with previous reports that did not find differences in locomotor activity in the OF test in aged asphyctic rats compared to control aged rats (Weitzdoerfer et al., 2004a,b). However, in contrast to our results, Weitzdoerfer et al. (2004a) reported increased anxiety levels in aged asphyctic animals in the EPM test. This discrepancy could be attributable to the following methodological differences between both studies: (a) In the study of Weitzdoerfer et al. (2004a), rats were submitted to 20 min of perinatal asphyxia instead of the 19 min used in the present study. It is well known that survival rate is drastically reduced and CNS damage increases with the duration of asphyxia (Loidl et al., 1994; Capani et al., 1997, 2009; Saraceno et al., 2012); (b) Weitzdoerfer et al. (2004a) used female rats, while only male rats were used in the present study. There are well known sex differences in basal emotional reactivity in the EPM and other anxiety tests; (c) Weitzdoerfer et al. (2004a) assessed older asphyctic rats (24 months old) than those used in the present study (18 months old). Such older rats could show confounding factors (lower mobility, for example) that could affect the anxiety measurement in the EPM test; (d) Finally, Weitzdoerfer et al. (2004a) measured some unfrequent behaviors to operationalize anxiety-related behaviors in the EPM test, such as the number of entries and the time spent in a box located at the end of one of the closed arms.

On the other hand, our results showed that middle-aged rats exposed to life-long EE, regardless of the birth condition, reduced their horizontal locomotor activity, showed a faster habituation response, and displayed lower anxiety-levels than rats reared in SE (see Figures 4, 5). In accordance to our data, other authors have reported a decrease of spontaneous motor activity in rats living in an enriched environment (Falkenberg et al., 1992; Del Arco et al., 2007; Segovia et al., 2008a,b). The results from the present study seem to indicate that these differences in motor activity could be attributable to a faster habituation shown by animals reared in EE. This study also shows that life-long exposure to EE produced a strong decrease in anxiety levels in all experimental groups. This anxiolytic effect is in line with the results obtained by other research groups which have subjected rodents to EE for medium to prolonged periods of time (Chapillon et al., 1999; Leal-Galicia et al., 2008; Hughes and Collins, 2010).

Neither the birth condition nor the housing environment affected the performance of animals in the NORT (see Figure 6). Other reports have also found that recognition memory, evaluated in the NORT, was unimpaired in aged rats (Solas et al., 2010; Gamiz and Gallo, 2012; Rushaidhi et al., 2013). In these cited studies and in the present work, the interval between sample and choice trials employed was one hour or less. When the inter-trial interval was increased to 24 h, an age-related deficit in the NORT was found by some other authors (de Lima et al., 2005; Burke et al., 2010; Leite et al., 2011). Thus, it could be possible that a one-hour inter-trial interval was too short to detect a possible deleterious influence of perinatal asphyxia on recognition memory. We avoided employing longer inter-trial intervals due to a possible floor effect that could also mask differences between control and asphyctic groups. On the other hand, EE was not able to enhance recognition memory in control groups. In this case, a ceiling effect could have been reached. From the results of the present study, it seems that EE has a more profound impact on spatial learning memories (MWM) than on non-spatial memories (NORT), in middle-aged rats.

Regarding the brain modifications induced by EE that could explain the improvement of cognitive performance, it has been demonstrated that environmental stimulation improves spatial cognition by inducing hippocampal neuroplasticity (i.e., dentate granule cell neurogenesis and glial proliferation) and by reducing spontaneous apoptotic cell death in the hippocampus (Young et al., 1999). This enhanced plasticity was associated with the induction of growth factor expression by EE (Young et al., 1999). On the other hand, more recently, it has been described a beneficial effect of exposure to environmental enrichment in anxiety-related behaviors (measured in the EPM test) that was correlated with changes in brain-derived neurotrophic factor (BDNF) expression in the central amygdala, hippocampus and the caudate putamen (Ravenelle et al., 2014).

## Concluding remarks

Our results indicate that perinatal asphyxia affects cognitive processes during aging, particularly spatial learning and reference and working memory. In addition, we also demonstrated that life-long exposure to EE is associated with reduced anxiety, faster habituation response to a novel environment, and a better spatial learning performance in middle-aged rats. Furthermore, life-long exposure to EE was able to counteract the spatial learning impairment, and the reference and working memory deficits of middle-aged asphyctic rats. These results support the importance of environmental stimulation across the lifespan to prevent cognitive deficits induced by perinatal asphyxia.

### Conflict of interest statement

The authors declare that the research was conducted in the absence of any commercial or financial relationships that could be construed as a potential conflict of interest.

## References

[B1] Adcock L. M. Papile L. A. (2008). Perinatal asphyxia, in Manual of Neonatal Care, eds Cloherty J. P. Eichenwald E. C. Stark A. R. (Philadelphia, PA: Lippincott Williams & Wilkins), 518–528.

[B2] Bennett J. C. McRae P. A. Levy L. J. Frick K. M. (2006). Long-term continuous, but not daily, environmental enrichment reduces spatial memory decline in aged male mice. Neurobiol. Learn. Mem. 85, 139–152. 10.1016/j.nlm.2005.09.003 16256380

[B3] Bjelke B. Andersson K. Ogren S. O. Bolme P. (1991). Asphyctic lesion: proliferation of tyrosine hydroxylase-immunoreactive nerve cell bodies in the rat substantia nigra and functional changes in dopamine neurotransmission. Brain Res. 543, 1–9. 10.1016/0006-8993(91)91041-X 1675922

[B4] Bruel-Jungerman E. Laroche S. Rampon C. (2005). New neurons in the dentate gyrus are involved in the expression of enhanced long-term memory following environmental enrichment. Eur. J. Neurosci. 21, 513–521. 10.1111/j.1460-9568.2005.03875.x 15673450

[B5] Burke S. N. Wallace J. L. Nematollahi S. Uprety A. R. Barnes C. A. (2010). Pattern separation deficits may contribute to age-associated recognition impairments. Behav. Neurosci. 124, 559–573. 10.1037/a0020893 20939657 PMC3071152

[B6] Cannon M. Jones P. B. Murray R. M. (2002). Obstetric complications and schizophrenia: historical and meta-analytic review. Am. J. Psychiatry 159, 1080–1092. 10.1176/appi.ajp.159.7.1080 12091183

[B7] Capani F. Loidl C. F. Aguirre F. Piehl L. Facorro G. Hager A.. (2001). Changes in reactive oxygen species (ROS) production in rat brain during global perinatal asphyxia: an ESR study. Brain Res. 914, 204–207. 10.1016/S0006-8993(01)02781-0 11578613

[B8] Capani F. Loidl C. F. Piehl L. L. Facorro G. De Paoli T. Hager A. (2003). Long term production of reactive oxygen species during perinatal asphyxia in the rat central nervous system: effects of hypothermia. Int. J. Neurosci. 113, 641–654. 10.1080/00207450390200099 12745625

[B9] Capani F. Loidl F. Lopez-Costa J. J. Selvin-Testa A. Saavedra J. P. (1997). Ultrastructural changes in nitric oxide synthase immunoreactivity in the brain of rats subjected to perinatal asphyxia: neuroprotective effects of cold treatment. Brain Res. 775, 11–23. 10.1016/S0006-8993(97)00714-2 9439823

[B10] Capani F. Saraceno G. E. Botti V. Aon-Bertolino L. de Oliveira D. M. Barreto G.. (2009). Protein ubiquitination in postsynaptic densities after hypoxia in rat neostriatum is blocked by hypothermia. Exp. Neurol. 219, 404–413. 10.1016/j.expneurol.2009.06.007 19555686

[B11] Chapillon P. Manneche C. Belzung C. Caston J. (1999). Rearing environmental enrichment in two inbred strains of mice: 1. Effects on emotional reactivity. Behav. Genet. 29, 41–46. 10.1023/A:1021437905913 10371757

[B12] Dahlqvist P. Ronnback A. Bergstrom S. A. Soderstrom I. Olsson T. (2004). Environmental enrichment reverses learning impairment in the Morris water maze after focal cerebral ischemia in rats. Eur. J. Neurosci. 19, 2288–2298. 10.1111/j.0953-816X.2004.03248.x 15090055

[B13] Del Arco A. Segovia G. Garrido P. de Blas M. Mora F. (2007). Stress, prefrontal cortex and environmental enrichment: studies on dopamine and acetylcholine release and working memory performance in rats. Behav. Brain Res. 176, 267–273. 10.1016/j.bbr.2006.10.006 17097747

[B14] de Lima M. N. Laranja D. C. Caldana F. Bromberg E. Roesler R. Schroder N. (2005). Reversal of age-related deficits in object recognition memory in rats with l-deprenyl. Exp. Gerontol. 40, 506–511. 10.1016/j.exger.2005.03.004 15935594

[B15] Duffy S. N. Craddock K. J. Abel T. Nguyen P. V. (2001). Environmental enrichment modifies the PKA-dependence of hippocampal LTP and improves hippocampus-dependent memory. Learn. Mem. 8, 26–34. 10.1101/lm.36301 11160761 PMC311356

[B16] Ennaceur A. (2010). One-trial object recognition in rats and mice: methodological and theoretical issues. Behav. Brain Res. 215, 244–254. 10.1016/j.bbr.2009.12.036 20060020

[B17] Ennaceur A. Delacour J. (1988). A new one-trial test for neurobiological studies of memory in rats. 1: behavioral data. Behav. Brain Res. 31, 47–59. 10.1016/0166-4328(88)90157-X 3228475

[B18] Falkenberg T. Mohammed A. K. Henriksson B. Persson H. Winblad B. Lindefors N. (1992). Increased expression of brain-derived neurotrophic factor mRNA in rat hippocampus is associated with improved spatial memory and enriched environment. Neurosci. Lett. 138, 153–156. 10.1016/0304-3940(92)90494-R 1407655

[B19] Galeano P. Blanco Calvo E. Madureira de Oliveira D. Cuenya L. Kamenetzky G. V. Mustaca A. E.. (2011). Long-lasting effects of perinatal asphyxia on exploration, memory and incentive downshift. Int. J. Dev. Neurosci. 29, 609–619. 10.1016/j.ijdevneu.2011.05.002 21640811

[B20] Galeano P. Martino Adami P. V. Do Carmo S. Blanco E. Rotondaro C. Capani F.. (2014). Longitudinal analysis of the behavioral phenotype in a novel transgenic rat model of early stages of Alzheimer's disease. Front. Behav. Neurosci. 8:321. 10.3389/fnbeh.2014.00321 25278855 PMC4165352

[B21] Galeano P. Romero J. I. Luque-Rojas M. J. Suarez J. Holubiec M. I. Bisagno V.. (2013). Moderate and severe perinatal asphyxia induces differential effects on cocaine sensitization in adult rats. Synapse 67, 553–567. 10.1002/syn.21660 23447367

[B22] Gamiz F. Gallo M. (2012). Spontaneous object recognition memory in aged rats: Complexity versus similarity. Learn. Mem. 19, 444–448. 10.1101/lm.027003.112 22984281

[B23] Hughes R. N. Collins M. A. (2010). Enhanced habituation and decreased anxiety by environmental enrichment and possible attenuation of these effects by chronic alpha-tocopherol (vitamin E) in aging male and female rats. Pharmacol. Biochem. Behav. 94, 534–542. 10.1016/j.pbb.2009.11.008 19941885

[B24] Ickes B. R. Pham T. M. Sanders L. A. Albeck D. S. Mohammed A. H. Granholm A. C. (2000). Long-term environmental enrichment leads to regional increases in neurotrophin levels in rat brain. Exp. Neurol. 164, 45–52. 10.1006/exnr.2000.7415 10877914

[B25] Iuvone L. Geloso M. C. Dell'Anna E. (1996). Changes in open field behavior, spatial memory, and hippocampal parvalbumin immunoreactivity following enrichment in rats exposed to neonatal anoxia. Exp. Neurol. 139, 25–33. 10.1006/exnr.1996.0077 8635565

[B26] Kempermann G. Gast D. Gage F. H. (2002). Neuroplasticity in old age: sustained fivefold induction of hippocampal neurogenesis by long-term environmental enrichment. Ann. Neurol. 52, 135–143. 10.1002/ana.10262 12210782

[B27] Komitova M. Perfilieva E. Mattsson B. Eriksson P. S. Johansson B. B. (2006). Enriched environment after focal cortical ischemia enhances the generation of astroglia and NG2 positive polydendrocytes in adult rat neocortex. Exp. Neurol. 199, 113–121. 10.1016/j.expneurol.2005.12.007 16427625

[B28] Leal-Galicia P. Castaneda-Bueno M. Quiroz-Baez R. Arias C. (2008). Long-term exposure to environmental enrichment since youth prevents recognition memory decline and increases synaptic plasticity markers in aging. Neurobiol. Learn. Mem. 90, 511–518. 10.1016/j.nlm.2008.07.005 18675926

[B29] Leggio M. G. Mandolesi L. Federico F. Spirito F. Ricci B. Gelfo F.. (2005). Environmental enrichment promotes improved spatial abilities and enhanced dendritic growth in the rat. Behav. Brain Res. 163, 78–90. 10.1016/j.bbr.2005.04.009 15913801

[B30] Leite M. R. Wilhelm E. A. Jesse C. R. Brandao R. Nogueira C. W. (2011). Protective effect of caffeine and a selective A2A receptor antagonist on impairment of memory and oxidative stress of aged rats. Exp. Gerontol. 46, 309–315. 10.1016/j.exger.2010.11.034 21122814

[B31] Lewis S. W. Murray R. M. (1987). Obstetric complications, neurodevelopmental deviance, and risk of schizophrenia. J. Psychiatr. Res. 21, 413–421. 10.1016/0022-3956(87)90088-4 3326936

[B32] Loidl C. F. Herrera-Marschitz M. Andersson K. You Z. B. Goiny M. O'Connor W. T.. (1994). Long-term effects of perinatal asphyxia on basal ganglia neurotransmitter systems studied with microdialysis in rat. Neurosci. Lett. 175, 9–12. 10.1016/0304-3940(94)91065-0 7970219

[B33] Lores-Arnaiz S. Bustamante J. Arismendi M. Vilas S. Paglia N. Basso N.. (2006). Extensive enriched environments protect old rats from the aging dependent impairment of spatial cognition, synaptic plasticity and nitric oxide production. Behav. Brain Res. 169, 294–302. 10.1016/j.bbr.2006.01.016 16513188

[B34] Lores-Arnaiz S. Bustamante J. Czernizyniec A. Galeano P. Gonzalez Gervasoni M. Rodil Martinez A.. (2007). Exposure to enriched environments increases brain nitric oxide synthase and improves cognitive performance in prepubertal but not in young rats. Behav. Brain Res. 184, 117–123. 10.1016/j.bbr.2007.06.024 17675170

[B35] McGuire W. (2006). Perinatal asphyxia. Clin. Evid. 511–519. 16973024

[B36] Mora F. Segovia G. del Arco A. (2007). Aging, plasticity and environmental enrichment: structural changes and neurotransmitter dynamics in several areas of the brain. Brain Res. Rev. 55, 78–88. 10.1016/j.brainresrev.2007.03.011 17561265

[B37] Nithianantharajah J. Hannan A. J. (2006). Enriched environments, experience-dependent plasticity and disorders of the nervous system. Nat. Rev. Neurosci. 7, 697–709. 10.1038/nrn1970 16924259

[B38] Obiang P. Maubert E. Bardou I. Nicole O. Launay S. Bezin L.. (2011). Enriched housing reverses age-associated impairment of cognitive functions and tPA-dependent maturation of BDNF. Neurobiol. Learn. Mem. 96, 121–129. 10.1016/j.nlm.2011.03.004 21440650

[B39] Paylor R. Morrison S. K. Rudy J. W. Waltrip L. T. Wehner J. M. (1992). Brief exposure to an enriched environment improves performance on the Morris water task and increases hippocampal cytosolic protein kinase C activity in young rats. Behav. Brain Res. 52, 49–59. 10.1016/S0166-4328(05)80324-9 1472287

[B40] Pereira L. O. Arteni N. S. Petersen R. C. da Rocha A. P. Achaval M. Netto C. A. (2007). Effects of daily environmental enrichment on memory deficits and brain injury following neonatal hypoxia-ischemia in the rat. Neurobiol. Learn. Mem. 87, 101–108. 10.1016/j.nlm.2006.07.003 16931063

[B41] Pereira L. O. Nabinger P. M. Strapasson A. C. Nardin P. Goncalves C. A. Siqueira I. R.. (2009). Long-term effects of environmental stimulation following hypoxia-ischemia on the oxidative state and BDNF levels in rat hippocampus and frontal cortex. Brain Res. 1247, 188–195. 10.1016/j.brainres.2008.10.017 18992724

[B42] Pereira L. O. Strapasson A. C. Nabinger P. M. Achaval M. Netto C. A. (2008). Early enriched housing results in partial recovery of memory deficits in female, but not in male, rats after neonatal hypoxia-ischemia. Brain Res. 1218, 257–266. 10.1016/j.brainres.2008.04.010 18514167

[B43] Rampon C. Tang Y. P. Goodhouse J. Shimizu E. Kyin M. Tsien J. Z. (2000). Enrichment induces structural changes and recovery from nonspatial memory deficits in CA1 NMDAR1-knockout mice. Nat. Neurosci. 3, 238–244. 10.1038/72945 10700255

[B44] Ravenelle R. Santolucito H. B. Byrnes E. M. Byrnes J. J. Donaldson S. T. (2014). Housing environment modulates physiological and behavioral responses to anxiogenic stimuli in trait anxiety male rats. Neuroscience 270, 76–87. 10.1016/j.neuroscience.2014.03.060 24713371 PMC4047719

[B45] Rice III J. E. Vannucci R. C. Brierley J. B. (1981). The influence of immaturity on hypoxic-ischemic brain damage in the rat. Ann. Neurol. 9, 131–141. 10.1002/ana.410090206 7235629

[B46] Rosenzweig M. R. Bennett E. L. (1996). Psychobiology of plasticity: effects of training and experience on brain and behavior. Behav. Brain Res. 78, 57–65. 10.1016/0166-4328(95)00216-2 8793038

[B47] Rushaidhi M. Zhang H. Liu P. (2013). Effects of prolonged agmatine treatment in aged male Sprague-Dawley rats. Neuroscience 234, 116–124. 10.1016/j.neuroscience.2013.01.004 23318245

[B48] Sampedro-Piquero P. Begega A. Arias J. L. (2014a). Increase of glucocorticoid receptor expression after environmental enrichment: relations to spatial memory, exploration and anxiety-related behaviors. Physiol. Behav. 129, 118–129. 10.1016/j.physbeh.2014.02.048 24582669

[B49] Sampedro-Piquero P. De Bartolo P. Petrosini L. Zancada-Menendez C. Arias J. L. Begega A. (2014b). Astrocytic plasticity as a possible mediator of the cognitive improvements after environmental enrichment in aged rats. Neurobiol. Learn. Mem. 114, 16–25. 10.1016/j.nlm.2014.04.002 24727294

[B50] Sampedro-Piquero P. Zancada-Menendez C. Begega A. Rubio S. Arias J. L. (2013). Effects of environmental enrichment on anxiety responses, spatial memory and cytochrome c oxidase activity in adult rats. Brain Res. Bull. 98, 1–9. 10.1016/j.brainresbull.2013.06.006 23831916

[B51] Saraceno G. E. Bertolino M. L. Galeano P. Romero J. I. Garcia-Segura L. M. Capani F. (2010). Estradiol therapy in adulthood reverses glial and neuronal alterations caused by perinatal asphyxia. Exp. Neurol. 223, 615–622. 10.1016/j.expneurol.2010.02.010 20206165

[B52] Saraceno G. E. Castilla R. Barreto G. E. Gonzalez J. Kolliker-Frers R. A. Capani F. (2012). Hippocampal dendritic spines modifications induced by perinatal asphyxia. Neural Plast. 2012, 873532. 10.1155/2012/873532 22645692 PMC3356716

[B53] Segovia G. Del Arco A. de Blas M. Garrido P. Mora F. (2008a). Effects of an enriched environment on the release of dopamine in the prefrontal cortex produced by stress and on working memory during aging in the awake rat. Behav. Brain Res. 187, 304–311. 10.1016/j.bbr.2007.09.024 17977609

[B54] Segovia G. Del Arco A. Garrido P. de Blas M. Mora F. (2008b). Environmental enrichment reduces the response to stress of the cholinergic system in the prefrontal cortex during aging. Neurochem. Int. 52, 1198–1203. 10.1016/j.neuint.2007.12.007 18242778

[B55] Solas M. Aisa B. Mugueta M. C. Del Rio J. Tordera R. M. Ramirez M. J. (2010). Interactions between age, stress and insulin on cognition: implications for Alzheimer's disease. Neuropsychopharmacology 35, 1664–1673. 10.1038/npp.2010.13 20182419 PMC3055481

[B56] Sun H. Zhang J. Zhang L. Liu H. Zhu H. Yang Y. (2010). Environmental enrichment influences BDNF and NR1 levels in the hippocampus and restores cognitive impairment in chronic cerebral hypoperfused rats. Curr. Neurovasc. Res. 7, 268–280. 10.2174/156720210793180819 20854252

[B57] Van de Berg W. D. Blokland A. Cuello A. C. Schmitz C. Vreuls W. Steinbusch H. W.. (2000). Perinatal asphyxia results in changes in presynaptic bouton number in striatum and cerebral cortex-a stereological and behavioral analysis. J. Chem. Neuroanat. 20, 71–82. 10.1016/S0891-0618(00)00078-8 11074345

[B58] van Handel M. Swaab H. de Vries L. S. Jongmans M. J. (2007). Long-term cognitive and behavioral consequences of neonatal encephalopathy following perinatal asphyxia: a review. Eur. J. Pediatr. 166, 645–654. 10.1007/s00431-007-0437-8 17426984 PMC1914268

[B59] van Praag H. Kempermann G. Gage F. H. (2000). Neural consequences of environmental enrichment. Nat. Rev. Neurosci. 1, 191–198. 10.1038/35044558 11257907

[B60] Vorhees C. V. Williams M. T. (2006). Morris water maze: procedures for assessing spatial and related forms of learning and memory. Nat. Protoc. 1, 848–858. 10.1038/nprot.2006.116 17406317 PMC2895266

[B61] Weitzdoerfer R. Gerstl N. Hoeger H. Mosgoeller W. Dreher W. Engidawork E.. (2002). Long-term sequelae of perinatal asphyxia in the aging rat. Cell. Mol. Life Sci. 59, 519–526. 10.1007/s00018-002-8443-5 11964129 PMC11337550

[B62] Weitzdoerfer R. Gerstl N. Pollak D. Hoeger H. Dreher W. Lubec G. (2004a). Long-term influence of perinatal asphyxia on the social behavior in aging rats. Gerontology 50, 200–205. 10.1159/000078348 15258424

[B63] Weitzdoerfer R. Pollak A. Lubec B. (2004b). Perinatal asphyxia in the rat has lifelong effects on morphology, cognitive functions, and behavior. Semin. Perinatol. 28, 249–256. 10.1053/j.semperi.2004.08.001 15565784

[B64] Young D. Lawlor P. A. Leone P. Dragunow M. During M. J. (1999). Environmental enrichment inhibits spontaneous apoptosis, prevents seizures and is neuroprotective. Nat. Med. 5, 448–453. 10.1038/7449 10202938

