# Supplementary material for: Life-long environmental enrichment counteracts spatial learning, reference and working memory deficits in middle-aged rats subjected to perinatal asphyxia
Source: Front Behav Neurosci. 2015 Jan 5;8:406. doi: 10.3389/fnbeh.2014.00406 (PMC4283640; doi:10.3389/fnbeh.2014.00406)
Supplement: Supplementary file 1 [file DataSheet1.DOC]

**Life-long environmental enrichment counteracts spatial learning, reference and working memory deficits in middle-aged rats subjected to perinatal asphyxia**

**Supplementary material**

**Pablo Galeano^1,2†*^, Eduardo Blanco^1,3,4†^, Tamara M. A. Logica Tornatore^1^, Juan I. Romero^1^, Mariana I. Holubiec^1^, Fernando Rodríguez de Fonseca^3^, Francisco Capani^1^**

^1^*Instituto de Investigaciones Cardiológicas “Prof. Dr. Alberto C. Taquini” (ININCA), Facultad de Medicina, UBA-CONICET, Buenos Aires, Argentina.*

^2^*Instituto de Investigaciones Bioquímicas de Buenos Aires, Fundación Instituto Leloir, IIBBA (CONICET), Ciudad Autónoma de Buenos Aires, Argentina.*

*^3^Laboratorio de Investigación, Instituto de Investigación Biomédica (IBIMA), Universidad de Málaga - Hospital Regional Universitario de Málaga (UGC Salud Mental), Málaga, Spain.*

^4^*Departamento de Psicobiología y Metodología de las Ciencias del Comportamiento, Facultad de Psicología, Universidad de Málaga, Instituto de Investigación Biomédica (IBIMA), Málaga, Spain.*

**^†^These two authors contributed equally to this work.**

Running title: **Environmental enrichment and perinatal asphyxia**

**^*^Correspondence:** Dr. Pablo Galeano, Instituto de Investigaciones Bioquímicas de Buenos Aires, Fundación Instituto Leloir, IIBBA (CONICET), Av. Patricias Argentinas 435, C1405BWE, Ciudad Autónoma de Buenos Aires.

[pgaleano@leloir.org.ar](mailto:pgaleano@leloir.org.ar)

**Supplementary material**

*1. Statistical analysis of the swimming speed in the spatial learning task*

To determine if groups differed in swimming speed during the spatial learning task, one-way ANOVA tests were carried out for each day of training. No differences were found in any of the days of training in the spatial learning task (day 1: *F*_(5, 66)_ = 0.752, *p* = n.s.; day 2: *F*_(5, 66)_ = 0.569, *p* = n.s.; day 3: *F*_(5, 66)_ = 1.616, *p* = n.s.; day 4: *F*_(5, 66)_ = 1.335, *p* = n.s.; day 5: *F*_(5, 66)_ = 0.690, *p* = n.s.).

*2. Statistical analysis of the swimming speed in the spatial working memory task*

Regarding swimming speed in the spatial working memory task, the three-way mixed ANOVA test indicated that neither the main effect of type of trial nor the main effect of environment nor the main effect of birth condition were significant (*F*_(1, 66)_ = 0.130, *p* = n.s.; *F*_(1, 66)_ = 0.209, *p* = n.s.; *F*_(2, 66)_ = 0.869, *p* = n.s., respectively). Furthermore, none of the interactions reached significance (environment x birth condition: *F*_(2, 66)_ = 1.025, *p* = n.s.; type of trial x environment: *F*_(1, 66)_ = 1.506, *p* = n.s.; type of trial x birth condition: *F*_(2, 66)_ = 0.950, *p* = n.s.; type of trial x birth condition x environment: *F*_(2, 66)_ = 0.872, *p* = n.s.). Post-hoc pairwise comparisons did not reveal statistical significant differences in swimming speed between the sample and retention trials in any of the groups (*p* = n.s. for all cases), although a strong tendency was found in C+ rats reared in EE (*p =* 0.065).
